# Parcellation of the human substantia nigra based on anatomical connectivity to the striatum^☆^

**DOI:** 10.1016/j.neuroimage.2013.05.043

**Published:** 2013-11-01

**Authors:** Rumana Chowdhury, Christian Lambert, Raymond J. Dolan, Emrah Düzel

**Affiliations:** a Institute of Cognitive Neuroscience, University College London, London WC1N 3AR, UK; b Wellcome Trust Centre for Neuroimaging, University College London, London WC1N 3BG, UK; c Stroke and Dementia Research Centre, St. George's University of London, Cranmer Terrace, London SW17 0RE, UK; d Otto-von-Guericke-University Magdeburg, Institute of Cognitive Neurology and Dementia Research, Leipziger Str. 44, 39120 Magdeburg, Germany; e German Center for Neurodegenerative Diseases (DZNE), Leipziger Str. 44, 39120 Magdeburg, Germany

**Keywords:** Connectivity, Diffusion-weighted imaging, Reward, Segmentation, Substantia nigra

## Abstract

Substantia nigra/ventral tegmental area (SN/VTA) subregions, defined by dopaminergic projections to the striatum, are differentially affected by health (e.g. normal aging) and disease (e.g. Parkinson's disease). This may have an impact on reward processing which relies on dopaminergic regions and circuits. We acquired diffusion tensor imaging (DTI) with probabilistic tractography in 30 healthy older adults to determine whether subregions of the SN/VTA could be delineated based on anatomical connectivity to the striatum. We found that a dorsomedial region of the SN/VTA preferentially connected to the ventral striatum whereas a more ventrolateral region connected to the dorsal striatum. These SN/VTA subregions could be characterised by differences in quantitative structural imaging parameters, suggesting different underlying tissue properties. We also observed that these connectivity patterns differentially mapped onto reward dependence personality trait. We show that tractography can be used to parcellate the SN/VTA into anatomically plausible and behaviourally meaningful compartments, an approach that may help future studies to provide a more fine-grained synopsis of pathological changes in the dopaminergic midbrain and their functional impact.

## Highlights

• We use DTI to segment the substantia nigra/ventral tegmental area (SN/VTA).
• Dorsomedial and ventrolateral SN/VTA regions were defined by striatal connectivity.
• R2* and fractional anisotropy values differed between SN/VTA subregions.
• Connectivity patterns differentially mapped onto a reward personality trait.

## Introduction

The anatomical topology of the SN/VTA can be defined by dopaminergic projections to the striatum. Classically, three dopaminergic regions are described: the substantia nigra pars compacta, the ventral tegmental area and the retrorubral field (Beckstead et al., 1979). The lack of clear boundaries between these regions in primates has led anatomists to refer to an ‘SN/VTA’ complex, divided into dorsal and ventral tiers which project mostly to the ventral and dorsal striatum respectively (Haber and Knutson, 2009; Haber et al., 2000). There is much interest in the use of neuroimaging to try to define the SN/VTA and its related anatomical circuits since this system is differentially affected by both health (e.g. normal aging) and disease (e.g. Parkinson's disease) (Fearnley and Lees, 1991; Vaillancourt et al., 2009, 2012) and plays an important role in many aspects of cognition including reward processing, learning and memory (Bäckman et al., 2006; Duzel et al., 2010; Shohamy and Adcock, 2010; Wise, 2004).

Magnetisation transfer (MT) imaging, which reflects the properties of bound protons (Henkelman et al., 2001; Wolff and Balaban, 1989), provides better grey/white matter contrast than the conventional MRI contrast and thus excellent visualisation of the SN/VTA (Helms et al., 2009). However, adequate MRI spatial resolution limits the ability to visualise subregions of the SN/VTA. Hence, dorsal and ventral tiers cannot be visually delineated on structural MR. An alternative approach is to use diffusion tensor imaging (DTI) and probabilistic tractography to segment structures based on their regional connectivity (Behrens and Johansen-Berg, 2005). DTI is a structural neuroimaging technique sensitive to the direction of water diffusion in tissues (Mori and Zhang, 2006; Moseley et al., 1990). This approach has been used to parcellate a range of cortical and subcortical structures (Behrens et al., 2003; Draganski et al., 2008; Johansen-Berg et al., 2008; Lambert et al., 2012; Menke et al., 2010; Rushworth et al., 2006). DTI-derived connectivity maps have close correspondence to the underlying cytoarchitectonics derived from post-mortem data (Klein et al., 2007; Seehaus et al., 2012) and relate to inter-individual differences in personality and behaviour (Cohen et al., 2009; Forstmann et al., 2011, 2012).

Previous studies have used different techniques to subdivide the SN/VTA (Lenglet et al., 2012; Menke et al., 2010). One such study used DTI whole-brain tractography and connectivity patterns to parcellate the substantia nigra into distinct regions resembling the SN pars compacta and pars reticulata and identified volumetric differences in the resulting subregions between healthy older adults and patients with Parkinson's disease (Menke et al., 2010). However, volumetric changes provide limited information about differences in underlying tissues. Quantitative MRI, on the other hand, can offer a window into the biological properties of tissues (Helms et al., 2009). For example, R2* (= 1/T2*) maps provide measures indicative of iron content (Martin, 2009; Martin et al., 2008) and DTI-derived fractional anisotropy (FA) maps are considered to be a marker of tissue organisation (Pfefferbaum et al., 2010; Vaillancourt et al., 2012). Using neuroimaging to better characterise the cellular properties of subregions may improve accuracy when distinguishing disease from non-disease states.

As neuroimaging techniques grow increasingly sophisticated, so too does the potential to better characterise the neural correlates of behaviour. Nigro-striatal anatomy is particularly relevant to reward processing, where dopamine plays a critical role (Salamone et al., 2005; Schultz et al., 1997; Wise, 2004). One measure of reward sensitivity is the reward dependence personality trait determined by the Tridimensional Personality Questionnaire (TPQ) (Cloninger, 1987a, 1987b, 1994; Gardini et al., 2009).The ventral striatum, which receives a huge dopaminergic input from the dorsal tier of the SN/VTA, is a core region of the reward system (Knutson and Greer, 2008; O'Doherty, 2004) and is linked to reward dependence (Cohen et al., 2009; Lebreton et al., 2009; Schreckenberger et al., 2008).

Therefore in the current study, we aimed to anatomically define, biologically characterise and assess the behavioural correlates of subregions of the SN/VTA based on differential anatomical connectivity to the dorsal and ventral striatum. To achieve this we obtained quantitative MRI and DTI images (MT, R2* and FA) from 30 healthy older adults. We used DTI tractography to parcellate the SN/VTA and used R2* and FA data to examine for different biophysical properties of the connectivity-based subregions. A critical question was whether the connectivity-derived networks between the SN/VTA and striatum were differentially associated with reward-related behaviour. To address this, we obtained a measure of a reward dependence personality trait from all individuals.

## Material and methods

### Participants

32 healthy older adults aged 65–75 years participated in this study. All participants were screened to ensure they did not have any of the following: neurological, psychiatric, cardiovascular, cerebrovascular or endocrinological disorders, metallic implants, or require more than one anti-hypertensive medication. All participants had a Mini-Mental State Examination score ≥ 28, Geriatric Depression Scale score ≤ 7 (a score > 11 would indicate depression) and a normal performance (within 1.5 SD of age-related norm) on a range of neuropsychological tests. All subjects had a normal neurological examination (performed by a neurologist R.C.) ensuring participants did not have concurrent undiagnosed neurological conditions. Written informed consent was obtained from all participants. The study received ethical approval from the North West London Research Ethics Committee 2. One participant was unable to tolerate DTI scanning. Tractography was unsuccessful in a further subject. Thus the final sample consisted of 30 right-handed healthy older adults (mean age 69.9 yrs, SD 3.26; 19 females).

Participants in the current study were selected from a larger sample of 42 healthy older adults who had participated in a previous study within the preceding six months. Preselection was based on an assessment of MT values of the SN/VTA in relation to a study examining the effects of SN/VTA structural integrity on episodic memory, full details of which have been published (Chowdhury et al., 2012). Briefly, we excluded 10 individuals with MT values of the SN/VTA scattered around the mean MT values of the group to select 16 participants with higher MT values and 16 with lower MT values. In the current study we note that MT values of the SN/VTA were normally distributed across participants (Kolmogorov–Smirnov and Shapiro–Wilk both p > 0.20). Note that this preselection criteria did not impact on the results of current the study (see Supplementary Results).

### Tridimensional Personality Questionnaire

Each participant completed the Tridimensional Personality Questionnaire (TPQ) (Cloninger, 1987a, 1987b). This self-report questionnaire consists of 100 true-false items measuring 3 personality traits: novelty-seeking, harm-avoidance and reward-dependence.

### Image acquisition

All MRI images were acquired using a 3.0 T Trio MRI scanner (Siemens) using a 32-channel head coil.

#### Anatomical MRI acquisition

A structural multi-parameter map protocol employing a 3D multi-echo fast low angle shot (FLASH) sequence at 1 mm isotropic resolution was used to acquire MT weighted (echo time, TE, 2.2–14.70 ms, repetition time, TR, 23.7 ms, flip angle, FA, 6°), proton density weighted (TE 2.2–19.7 ms, TR 23.7 ms, FA 6°) and T1 weighted (TE 2.2–14.7 ms, TR 18.7 ms, FA 20°) images (Helms et al., 2008b). B1 mapping (TE 37.06 and 55.59 ms, TR 500 ms, FA 230:− 10:130°, 4 mm isotropic resolution) was acquired to correct the T1 maps for inhomogeneities in the transmit radiofrequency field (Lutti et al., 2010). A double-echo FLASH sequence (TE1 10 ms, TE2 12.46 ms, 3 × 3 × 2 mm resolution and 1 mm gap) was used to measure local field inhomogeneities and correct for the image distortions in the B1 mapping data. MT maps were used to manually define the SN/VTA. Note although we did not use the T1 and proton density maps for any analyses, we report the sequence protocols here as they are crucial for estimating the MT maps (Helms et al., 2008b).

#### DTI acquisition

We acquired diffusion weighted images using a spin-echo echoplanar imaging (EPI) sequence, with twice refocused diffusion-encoding to reduce eddy-current-induced distortions (Reese et al., 2003). Amplitudes of diffusion-encoding gradients were calibrated for unbiased measurement of diffusion directions and improved fibre tracking (Nagy et al., 2007). We acquired 75 axial slices (whole brain to mid-pons) in an interleaved order [1.7 mm isotropic resolution; image matrix = 96 × 96, field of view = 220 × 220 mm^2^, slice thickness = 1.7 mm with no gap between slices, repetition time (TR) = 170 ms, echo time (TE) = 103 ms, asymmetric echo shifted forward by 24 phase-encoding (PE) lines, readout bandwidth (BW) = 2003 Hz/pixel] for 61 images with unique diffusion encoding directions. The first seven reference images were acquired with a b-value of 100 s/mm^2^, the remaining 61 images with a b-value of 1000 s/mm^2^ (Nagy et al., 2007). Two DTI sets were acquired with identical parameters except that the second was acquired with a reversed k-space readout direction to allow removal of susceptibility artefacts post-processing (Andersson et al., 2003). Since the SN/VTA was a major region of interest, we optimised the quality of our images by using pulse-gating to minimise pulsation artefact within the brainstem. The total data acquisition protocol lasted approximately 40 min depending on each individual's heart rate.

### DTI preprocessing

FSL version 4.1.4 was used for DTI pre-processing. First, images were eddy current corrected. Correction for susceptibility artefacts was performed as previously described (Andersson et al., 2003). The low b images were averaged and used to generate a brain mask for skull stripping, which was performed using SPM8 New Segment and ImCalc. The skull stripped brain was manually checked for errors prior to further processing, and corrections performed where necessary. Initial estimation of tensors was performed using DTI fit allowing fractional anisotropy (FA) to be calculated, and all the results were visually checked prior to full estimation of the diffusion parameters. BEDPOSTX was used to estimate the probability distributions of two fiber populations at each voxel (Behrens et al., 2007). Finally, FSLs non-linear registration algorithm FNIRT was used to generate two warp fields to allow sampling between diffusion and structural space, and the results of these were manually checked for all individuals to ensure optimal alignment.

### DTI tractography

All tractography was performed in each individual's native space. Tractography analysis was carried out from all voxels in each subject's anatomically-defined SN/VTA ROI, separately for right and left. The anatomical delineation is outlined later in the methods. To avoid erroneous tractography results, we created individual subject exclusion masks using ITK-SNAP (Yushkevich et al., 2006). The ventricles and CSF spaces were automatically defined using the “snake” function, and particular attention was paid to manually refine the region surrounding the cerebral peduncle and medial wall of the temporal lobe. Tractography was run using FSL's probtrackX software (Behrens et al., 2007). Each voxel was sampled 5000 times with a burn in of 1000, curvature threshold of 0.2, modelling two fibres per voxel, utilising the previously calculated warp fields.

#### SN/VTA (tractography seed mask)

**Fig. 1:**
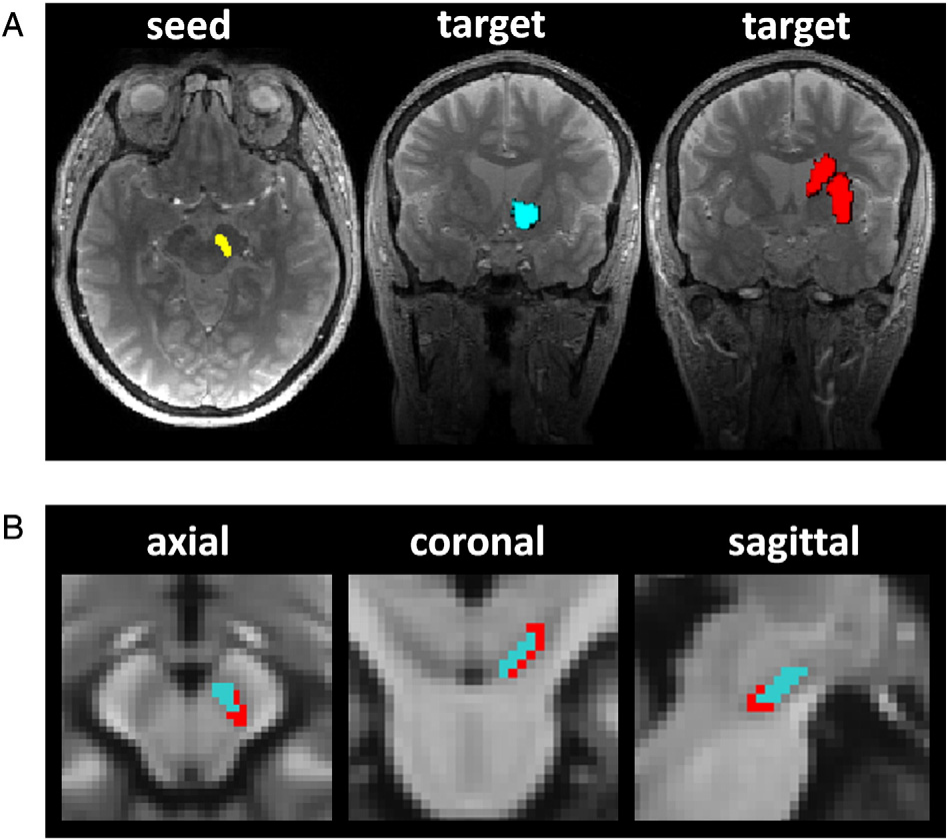
Parcellation of the substantia nigra based on connectivity to the striatum. A: Single subject example of seed region for tractography (SN/VTA) and the ventral striatum (blue) and dorsal striatum target regions (red). B: The substantia nigra was parcellated into two overlapping subregions based on tractography-based connectivity to the striatum. The dorsomedial-SN (blue) connected to the ventral striatum, whereas a more ventrolateral-SN subregion (red) connected to the dorsal striatum. Images are of a group probability map thresholded at 50% overlap, overlayed on a group-average MT image in the axial, coronal and sagittal planes from left to right.

The medial and lateral boundaries of the SN/VTA were defined on each subjects' MT-weighted image where it is easily distinguishable from the surrounding tissues due to its bright grey colour in contrast to the adjacent cerebral peduncle. This region was manually defined by R.C. on every visible slice (between seven to ten slices) as per Duzel et al. (2008)) using MRIcro (Rorden, 2000). Ten randomly selected SN/VTA ROIs were segmented by a second trained individual (C.L.) and showed high inter-rater reliability, (Intraclass correlation coefficient = 0.87, p < 0.0005, 95% confidence interval 0.129–0.973; calculated using a two-way random absolute agreement model in SPSS). Fig. 1A shows a single-slice single-subject example of the right SN/VTA seed.

#### Striatum (tractography target masks)

To define the ventral striatum (nucleus accumbens) in our older cohort of subjects, we made a subject-derived mask for this region. We used Freesurfer's (version 4.5.0, http://surfer.nmr.mgh.harvard.edu/) automated recon-all pipeline to parcellate cortical and subcortical regions (Fischl et al., 2004). Each subjects' ventral striatum mask was visually inspected to ensure accurate segmentation. Subjects were excluded due to preprocessing errors (n = 4) or inaccurate segmentation after visual inspection (n = 3). For the remaining 23 participants, their ventral striatum masks were warped to MNI space using DARTEL in SPM8 (Ashburner, 2007) and then group-averaged and binarised. This average mask was then normalised to each individuals' native space (n = 30) using the inverse of the normalisation parameters. The aim of this approach was to obtain a target ventral striatum mask that was accurate and representative of our older cohort but the same size for each individual, hence we averaged the mask across the group. Thus we report DTI data for all 30 participants.

The dorsal striatum was defined using the caudate and putamen masks from the AAL toolbox (Tzourio-Mazoyer et al., 2002). The group-averaged ventral striatum mask was subtracted from this caudate-putamen mask to make a non-overlapping dorsal striatum mask. This MNI-space mask was then normalised to each individuals' native space using the inverse of the normalisation parameters. Fig. 1A shows a single-slice single-subject subject example of the right ventral and dorsal striatum target masks.

#### Quantitative tractography metrics

We generated ‘relative connectivity strength’ maps as per Forstmann et al. (2011), Forstmann et al. (2012)) using the following steps. For clarity, here we refer to the probabilistic index of connectivity (PICo), which is defined as the number of traces arriving at any given voxel from a tractography seed, and is equivalent to the term “samples” used by other authors. Step 1: Generate individual seed voxel PICo maps for every seed voxel. In each map, the voxel values represents the number of samples (from 0 to 5000) originating from the seed passing through a voxel, using probtrackX. Step 2: Generate individual ROI probability maps. First we calculated the maximum PICo value that occurred within the ROI of interest across all seed PICo maps. We then thresholded each individual seed PICo map at 0.02% of the maximum ROI PICo value. This threshold is consistent with that used by previous groups (Aron et al., 2007; Forstmann et al., 2011, 2012). The individual seed maps were combined so that the final value at each ROI voxel then becomes the maximum PICo for that specific voxel across every thresholded seed PICo map. Step 3: Generate “Relative Connectivity Strength” maps. The ROI probability maps were divided by the sum of all PICo values, such that the value at each voxel represents the maximum PICo for that voxel divided by the sum of all PICo values within that specific map. An average of these voxel values was calculated to obtain a single average connectivity strength value for each subregion.

#### Parcellating the SN/VTA

Using the tractography results from the SN/VTA region on each side, we subdivided each SN/VTA. This was achieved by defining, at each seed voxel, whether a connection survived thresholding to the dorsal and ventral striatum targets as defined above, generating an individual mask image for each SN/VTA subregion.

### Quantitative imaging metrics

To determine whether the subregions of the SN/VTA we defined based on connectivity to the dorsal and ventral striatum reflected regions with different underlying tissue properties, we used in-house code to extract R2* (1/T2*) quantitative maps for each subject (Helms et al., 2008a). R2* values are sensitive to iron content (Draganski et al., 2011; Martin, 2009; Martin et al., 2008). We also obtained FA maps (see above ‘DTI preprocessing’ section for details). DTI is based on the principle that in a non-homogeneous medium, water diffusion is restricted in certain directions (‘anisotropic’). A ‘diffusion ellipsoid’, characterised by eigenvalues (axes) and eigenvectors (orientations) can be estimated at every voxel (Basser et al., 1994). The ellipsoid shape relates to fractional anisotropy (FA) and the principle orientation is used for fibre tracking (Le Bihan and Johansen-Berg, 2012). FA values characterise the extent of water diffusion in every voxel with values ranging from zero (full isotropy) to one (full anisotropy), so providing a measure of the ‘structural organisation’ of both grey and white matter (Mori et al., 2008; Vaillancourt et al., 2009, 2012). Mathematically, FA values represent the standard deviation of the eigenvalues normalised by the tensor magnitude (Pierpaoli and Basser, 1996). We calculated FA and R2* values locally within the connectivity-based SN/VTA subregions.

It has previously been observed that the SN pars reticulata has significantly higher iron content than the SN pars compacta (Drayer et al., 1986). We therefore tested whether the R2* images alone, which reflect iron content, could be used to parcellate the SN/VTA, and whether these clusters had a significant impact on the observed biophysical differences in the connectivity-based parcellations. We tested three hierarchical hypotheses: 1) The SN/VTA can be sub-parcellated using R2* data alone. 2) If subregions in R2* data exist, do they significantly overlap with the observed connectivity based parcellations. 3) If they do not simply overlap, are the observed differences in the SN/VTA connectivity based parcellations R2* values merely due to differential sampling of these R2s clusters that weights the signal in one region more than the other. Using K-means clustering with randomised initiation and 1000 repetitions, the R2* data was used to cluster the SN/VTA into two subregions for every individual SN/VTA, and re-ordered across the group to ensure consistent inter-individual labelling.

### Statistical analysis

#### Imaging parameters within SN/VTA subregions

We performed within-subject comparisons of R2* and FA values between SN/VTA subregions. For R2* values we report paired t-tests. Where assumptions of normality were violated for FA values, we used the Wilcoxon Signed Ranks Test. The significance level was set at p < 0.0125 after Bonferroni correction for four tests (left and right R2* and FA). All p-values are two-tailed. To examine for cross-correlations between imaging parameters we report two-tailed Spearman's correlations (averaged for left and right).

#### Connectivity strength and personality

We used our relative connectivity strength maps to measure right and left SN/VTA-striatum connectivity strength in relation to reward dependence. We performed a median split of participants based on personality scores (separate analyses for reward dependence, novelty seeking and harm avoidance) and used a repeated measures ANCOVA with pathway connectivity strength (dorsomedial SN/VTA to ventral striatum/ventrolateral SN/VTA to dorsal striatum) as the within-subjects factor and personality group score (low/high) as the between-subjects factor. This approach allowed us to identify interactions which would indicate a dissociation between the two SN/VTA-striatum pathways with regards to their contribution to trait characteristics. Since these analyses included a between-subjects component, we included age, gender and total intracranial volume as covariates. For significant interactions, we followed this up with post hoc independent t-tests (two-tailed) and correlations across all participants. For correlations, we confirmed there were no outliers (all participants had connectivity strength z-scores − 3 < z < 3) and report two-tailed partial Spearman's correlations (controlling for age, gender and total intracranial volume) as has been recommended (Schwarzkopf et al., 2012).

## Results

### Connectivity-based parcellation of SN/VTA

Using DTI, we performed probabilistic tractography from a manually defined seed region—the SN/VTA. Subregions of the SN/VTA were then obtained based on connectivity to two target regions—the dorsal and ventral striatum. Visual inspection of the results in each subject revealed that a more dorsomedial region of the SN/VTA was defined by connectivity to the ventral striatum (hereafter referred to as dorsomedial-SN) whereas a more ventrolateral region of the SN/VTA was defined by connectivity to the dorsal striatum (hereafter referred to as ventrolateral-SN) (Fig. 1B). Mean volumes of these connectivity-based SN/VTA subregions are reported in Supplementary Table 1.

### Imaging parameter values of SN/VTA subregions

**Fig. 2:**
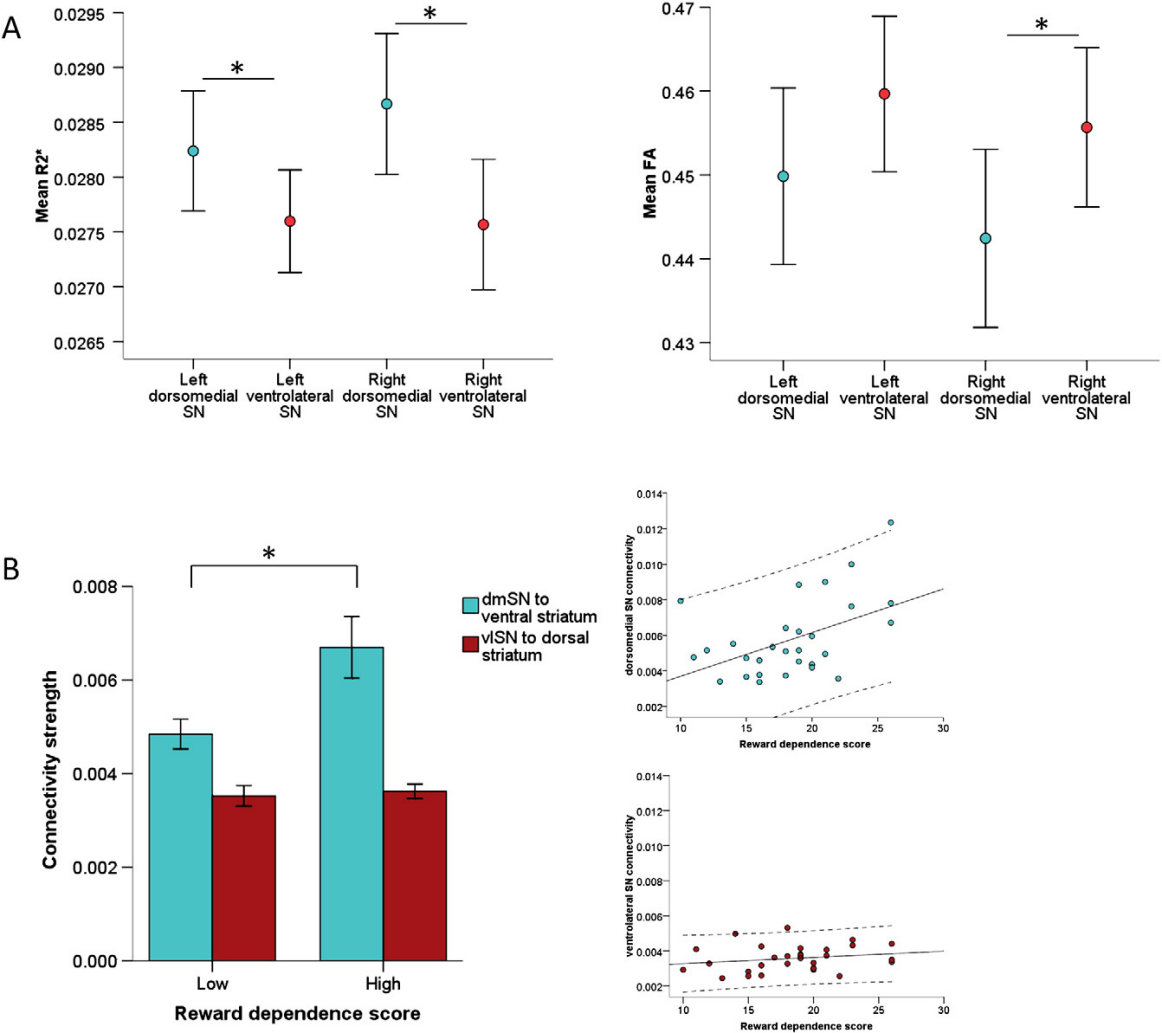
Imaging parameters and reward dependence are dissociated between SN/VTA-striatal pathways. A: Higher R2* values and lower fractional anisotropy (FA) values were evident in the dorsomedial-SN subregion defined by preferential connectivity to the ventral striatum (blue), compared to the ventrolateral-SN subregion defined by preferential connectivity to the dorsal striatum (red). Bars are ± 1SEM. * p < 0.0125 two-tailed. B: Higher connectivity strength between the dorsomedial-SN and ventral striatum, but not ventrolateral-SN and dorsal striatum, was associated with higher reward dependence personality scores. Results control for age, gender and total intracranial volume. dmSN = dorsomedial substantia nigra; vlSN = ventrolateral substantia nigra. * p < 0.05. On the scatter plots, each dot represents an individual, the solid line represents the linear regression and the dashed lines are 95% confidence intervals.

Our comparison of quantitative R2* maps and FA maps suggested different underlying tissue properties in the dorsomedial and ventrolateral subregions of the SN/VTA as defined on the basis of connectivity (Fig. 2A). R2* values were significantly higher in both the right and left dorsomedial-SN compared to the ventrolateral-SN (two-tailed paired t-tests, right: t = 4.52, p < 0.0005; left: t = 2.87, p = .008). In contrast, FA values were significantly lower in the right dorsomedial-SN compared to the ventrolateral-SN, with a similar trend in the left SN/VTA (two-tailed Wilcoxon Signed Ranks Test, right: Z = − 2.90, p = .004; left: Z = − 1.99; p = .047) (significance level p < 0.0125 after Bonferroni correction for four tests) (see Fig. S1 for individual pairings). This suggests higher iron content (indexed by R2*) and less structural organisation (indexed by FA) of the dorsomedial-SN compared to the ventrolateral-SN subregions that we defined.

We considered whether the pattern of imaging parameter values in the SN/VTA subregions could be due to cross-correlations between the parameters themselves. This would suggest that the MRI sequence properties rather than the underlying tissue biological properties could account for our results, for example, increased iron deposition can cause signal loss which in turn could influence DTI metrics (Pfefferbaum et al., 2010). However R2* did not correlate significantly with FA in either dorsomedial-SN (Spearman's Rho = 0.16, p = .397) or ventrolateral-SN (Spearman's Rho = 0.16, p = .408), therefore this could not explain our pattern of findings.

In a separate analysis of the R2* data using K-means clustering, we defined two SN/VTA subregions (Fig. S2). To characterise this further, in every individual, we calculated the overlap between the R2* clusters and the connectivity-based clusters and expressed this as a percentage of the connectivity-based cluster that was contained within the R2* cluster (results summarised in Table S2). This showed there was considerable overlap between the two R2* clusters and the dorsomedial and ventrolateral components of the SN/VTA. We next analysed if there were any significant differences between the volumes of clusters within each region i.e. was there a significant skew in R2* clusters towards the dorsomedial and ventrolateral subregions that could account for the observed R2* differences in the connectivity-based parcellations. We found a significant difference in the right SN/VTA (t(29) = 4.72, p = .001), with a mean difference of 4.8% in intra-cluster size between the dorsomedial and ventrolateral subregions, whereas there was no difference in the left SN/VTA (t(29) = 0.88, p = .384). Given that significant differences in R2* was observed in both the left and right SN/VTA connectivity-based parcellations, it follows that these differences cannot be simply due to differential sampling of R2* sub-clusters but rather an independent effect. It may, however, account for why a slightly greater difference in R2* values was observed between the right SN/VTA connectivity-based subregions.

### SN/VTA-striatal connectivity and reward dependence

To determine if there was dissociation between reward-related behaviour and connectivity strength between the two pathways we identified, we divided participants into those with a high or low reward dependence personality trait. We found that older adults with higher reward dependence scores had a greater difference of connectivity strength between the two right-sided pathways (ANCOVA controlling for age, gender and TIV: pathway* reward dependence -group interaction: F(1,25) = 4.91, p = .036; main effect pathway: F(1,25) = 0.04, p = .845; all interactions between pathway and covariates p > 0.2). Planned post hoc tests revealed this interaction arose because high reward dependent individuals had higher right dorsomedial SN-ventral striatal connectivity strength compared to those who were less reward dependent (independent t-test, t (28) = − 2.54, p = .017). In contrast, right ventrolateral SN-dorsal striatum connectivity strength did not differ between groups (t(28) = − 0.36, p = .721). There was a positive correlation across all participants between right dorsomedial SN-ventral striatal connectivity strength and reward dependence scores (partial Spearman's controlling for age, gender and TIV: rho = 0.41, p = .034), but no correlation with right ventrolateral SN-dorsal striatum connectivity strength (rho = 0.17, p = .392).

These findings were restricted to right-sided connectivity (ANCOVA controlling for age, gender and TIV: left-sided pathway*reward dependence-group interaction: F(1,25) = 0.48, p = .497). With regards to the other two personality measures, connectivity strength was similar for individuals with low and high novelty seeking scores (pathway*novelty seeking-group interaction, left: F(1,25) = 2.28, p = .143; right: F(1,25) = 0.04, p = .836) and harm avoidance scores (pathway*harm avoidance-group interaction, left: F(1,25) = 0.68, p = .422; right: F(1,25) = 3.08, p = .092), indicating the specificity of our findings to reward dependence.

## Discussion

Using DTI tractography, we show that the SN/VTA can be subdivided into dorsomedial and ventrolateral subregions based on preferential connectivity to the ventral and dorsal striatum respectively. These findings are in keeping with multiple tract tracing studies that demonstrate segregation of dorsal and ventral tiers of the SN/VTA with respect to a network of spiral cortico-striato-nigral loops (Haber and Knutson, 2009; Haber et al., 2000). Furthermore, the subregions we identified differed in their underlying tissue properties (indexed by quantitative MRI parameters) and the associated pathways to the striatum were differentially associated with a reward-dependence personality trait, lending further validity to our findings.

One previous study has used DTI tractography and a clustering approach to divide the SN/VTA into two distinct subregions (Menke et al., 2010). Our parcellation of the SN/VTA broadly corresponds with the regions found in that study, which the authors suggest distinguish between the pars compacta and pars reticulata. However, in our study we were interested in subdividing the SN/VTA based on its connectivity to the striatum to try to more closely mimic the known anatomical dominance of dopamine projections between these regions. Indeed a more practical division of the SN/VTA may be into dorsal and ventral tiers since the pars compacta and pars reticulata often protrude into one another (for a review see (Duzel et al., 2009). Interestingly, although our striatal target masks were non-overlapping, the SN/VTA subregions delineated by connectivity to the striatum did partially overlap. Despite this partial topographical overlap, we observed different imaging parameter values within these subregions suggesting that they were not only topographically separable but also had different tissue properties. We found that the dorsomedial SN/VTA subregion had higher R2* and lower FA values than the ventrolateral SN/VTA subregion, which may reflect higher iron content and reduced microstructural integrity respectively. These bilateral differences of R2* values within the connectivity-based SN/VTA subregions was independent from an alternative partially-overlapping axis of parcellation of the SN/VTA which we defined using R2* values. Differences in tissue properties in dopaminergic projections targeting the dorsal and ventral striatum are not surprising (Liss and Roeper, 2008). According to this view, the properties of dopaminergic neurons are not only determined by their connectivity, but there are also biochemical and physiological differences between dopamine neurons in different projection pathways (Liss and Roeper, 2008).

A key novel finding from our study is the demonstration of a behavioural dissociation between the pathways associated with the dorsal and ventral SN/VTA. We examined how connectivity strength was related to reward dependence since there is converging evidence that the ventral striatum is linked to this personality trait. Greater ventral striatum grey matter volume (Lebreton et al., 2009), metabolic activity (Schreckenberger et al., 2008) and connectivity to the prefrontal cortex (Cohen et al., 2009) are all associated greater reward dependence. As hypothesised, we found that higher connectivity strength between the dorsomedial SN/VTA and ventral striatum was associated with greater reward dependence, whereas there was no difference in ventrolateral SN-dorsal striatum connectivity between participants with high and low reward dependence. This relationship conforms to the predictions made based on existing evidence of the involvement of ventral striatum and its associated pathways in reward dependence, so lending behavioural validity to our segmentation approach. These findings also further extend our knowledge of the neural correlates of this personality trait. We note that in terms of structural correlates, the ventral striatum is strongly linked to reward dependence. However, with regards to neurotransmitters, studies relate reward dependence to the noradrenergic system rather than the dopaminergic system (Gardini et al., 2009). We acknowledge that DTI tractography is not a direct mapping of dopamine neurons but rather reflects the white matter tracts between regions and therefore we cannot know to what extent our connectivity measure relates to the pattern of striatal dopamine release.

We did not have a younger age group for comparison and are therefore unable to make age-specific interpretations. One interesting question for future research is whether healthy older individuals will show greater age-related change in the dorsomedial compared to ventrolateral subregion. This may be relevant given that histological studies show that older adults have greater dopamine neuron loss from dorsal SN/VTA (Fearnley and Lees, 1991). A previous DTI study showed age-related reduction of FA in manually-defined dorsal but not ventral SN subregions (Vaillancourt et al., 2012). A different pattern has been reported in Parkinson's disease where patients have reduced FA in the ventrolateral SN, in keeping with the known marked dopamine loss from this region (Vaillancourt et al., 2009). These studies, together with animal models, provide converging evidence that FA of the SN/VTA may be a marker of underlying dopaminergic architecture (Boska et al., 2007; Vaillancourt et al., 2009).

In our study we focussed on connectivity between the SN/VTA and striatum since these are major well-characterised anatomical projections. However, direct projections between the SN/VTA and other structures exist, for example with the prefrontal cortex (Kunzle, 1978) and hippocampus (Gasbarri et al., 1994). It would be interesting for future studies to attempt connectivity-based parcellation of the SN/VTA with other such structures in mind, although care will need to be taken to robustly identify these minor projections to more distant regions.

### Conclusions

In summary, we show that it is possible to define dorsomedial and ventrolateral subregions of the SN/VTA using DTI-based connectivity to the ventral and dorsal striatum. Combining this parcellation of the SN/VTA with a range of imaging parameters may help to better quantify midbrain changes both in health and disease. Finally, our segmentation schema produces outputs that can be linked to reward-related behaviour. This both lends validity to our findings and highlights one of the many important ways that DTI and its related metrics can be used to obtain a more fine-grained understanding of the neural correlates of behaviour.

## Funding

RC is supported by a Wellcome Trust Research Training Fellowship number WT088286MA. RD is supported by the Wellcome Trust, grant number 078865/Z/05/Z. The Wellcome Trust Centre for Neuroimaging is supported by core funding from the Wellcome Trust 091593/Z/10/Z.
