# Supplementary material for: Parcellation of the human substantia nigra based on anatomical connectivity to the striatum
Source: Neuroimage. 2013 Nov 1;81:191–8. doi: 10.1016/j.neuroimage.2013.05.043 (PMC3734352; doi:10.1016/j.neuroimage.2013.05.043)

**Supplementary Materials: Parcellation of the human substantia nigra based on anatomical connectivity to the striatum**

Rumana Chowdhury, Christian Lambert, Raymond J Dolan, Emrah Düzel

**Supplementary Results**

**Relationship between preselection criteria and study results**

Subregion differences in R2* and FA values were unrelated to MT group (repeated measures ANOVA with SN/VTA subregion (dorsal/ventral) as the within-subjects factor and MT group (low/high) as the between subjects factor: main effect of FA values of subregion = F(1,28) = 9.16, p = .005; FA by MT group interaction F(1,28) = 0.17, p = .688 ; main effect of R2* values of subregion F(1,28) = 28.1, p = .000; R2* by MT group interaction F(1,28) = 0.54, p = .467). The interaction between reward dependence and connectivity strength also persisted when MT was included as a covariate along with age, gender and TIV (F(1,24) = 4.60, p = .04) and there was no interaction with MT groups (F(1,24) = 0.21, p = .652). These results show that the preselection criteria based on MT values did not impact upon the results of the current study.

Table S1. Mean volumes of the substantia nigra subregions that resulted from tractography-based parcellation to the striatum (n = 30). Dorsomedial subregions were smaller than ventrolateral subregions (p < 0.0005).

|  | **Subregion** | **Volume** |
| --- | --- | --- |
|  |  | **Mean ± SD (mm3)** |
| **Left** | Dorsomedial | 180 ± 71 |
|  | Ventrolateral | 312 ± 57 |
| **Right** | Dorsomedial | 194 ± 60 |
|  | Ventrolateral | 291 ± 59 |

**Table S2. Overlap between the R2* clusters and the connectivity-based clusters, expressed as a percentage of the connectivity-based cluster that was contained within the R2* cluster**

|  | **Dorsomedial-SN** | | **Ventrolateral-SN** | |
| --- | --- | --- | --- | --- |
|  | **Left** | **Right** | **Left** | **Right** |
| **R2* Cluster 1** | 40% | 42% | 39% | 37% |
| **R2* Cluster 2** | 60% | 58% | 61% | 63% |

Figure S1 Plots showing individual FA and R2* values paired across SN subregions (n=30 per subregion). Blue circle = mean dorsomedial-SN value; red circle = mean ventrolateral-SN value

**Figure S2. SN/VTA parcellation using R2* data and K-means clustering. This single subject example shows the two clusters (white and brown).**


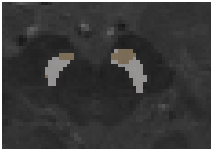

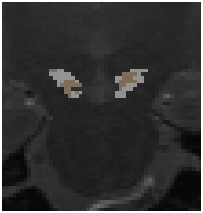

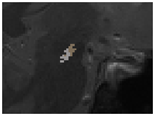

Supplement: Supplementary file 1 — Supplementary material [file mmc1.docx]
